# Cardio-Respiratory, Functional and Antalgic Effects of the Integrated Thermal Care Protocol After Breast Cancer Surgery

**DOI:** 10.3390/life15030374

**Published:** 2025-02-27

**Authors:** Giovanni Barassi, Stefania Spina, Francesco D’Alessandro, Loris Prosperi, Celeste Marinucci, Massimo Lombardi, Maurizio Panunzio, Andrea Santamato

**Affiliations:** 1 Castelnuovo della Daunia Thermal Medicine Center, 71034 Castelnuovo della Daunia, Italy; 2 Center for Physiotherapy, Rehabilitation and Re-Education (Ce.Fi.R.R.) Venue “G. d’Annunzio” University of Chieti-Pescara, 66100 Chieti, Italy; 3 Spasticity and Movement Disorders “ReSTaRt”, Physical Medicine and Rehabilitation Section, Department of Medical and Surgical Sciences, University of Foggia, 71122 Foggia, Italy; 4 Oncology Sector, “Teresa Masselli Mascia” Hospital, 71016 San Severo, Italy; 5 Responsible Research Hospital, 86100 Campobasso, Italy

**Keywords:** rehabilitation, physical therapy, balneotherapy, mineral waters, breast cancer, mastectomy, fatigue, pain, manual therapy, hydrotherapy

## Abstract

Background: In the cardio-respiratory rehabilitation field, thermal medicine represents an interesting complementary therapy approach. It can aid in complex medical contexts characterized by cardio-respiratory deficiency, functional limitation, and pain determined by the invasiveness of pharmacological and surgical treatments in combination with limited post-surgical physical activity. Methods: We investigated the evolution of cardio-respiratory and functional performances following the application of the Integrated Thermal Care (ITC) protocol in 11 mastectomized/quadrantectomized women (mean age of 54 years). The ITC protocol consisted of hydroponic treatments, steam inhalations treatment, hydrokinesitherapy, and manual treatments. Patients were assessed before and after a cycle of 1 h long treatment sessions, which were performed 5 days a week for 4 weeks. The outcomes were measured through the following scales and tests: Piper Fatigue Scale (PIPER), 6-Minute Walking Test (6MWT), Five Times Sit-to-Stand (5STS), Range of Arm Motion (ROM), Disability of the Arm–Shoulder–Hand Scale (DASH), and Numeric Pain Rating Scale (NPRS). Results: We found appreciable improvements in cardio-respiratory efficiency and in pain perception exemplified by a reduction of PIPER, 5STS, DASH, and NPRS values together with an increase in 6MWT and ROM values. Conclusions: We conclude that ITC is a promising rehabilitative tool to enhance cardio-respiratory and functional performance and reduce pain after mastectomy/quadrantectomy.

## 1. Introduction

Thermal medicine, often also called balneotherapy or spa treatments, is one of the oldest therapeutic approaches known to humanity, and it has been used since ancient times in the most disparate ways for a multitude of physical pathologies by relying on the natural healing properties that have always been attributed to thermal waters [1]. Despite this, to date, thermal medicine appears to be a curative approach that is still little understood in terms of mechanisms of action and significance of efficacy in its multiple uses [1], constituting an underrated complementary and alternative medical branch that could however guarantee an improvement in the state of health for large segments of the population. It is no coincidence that in Italy, some laws attribute to thermal medicine an important role as maintenance therapy in the field of community medicine [2,3].

Over the years, thermal medicine has repeatedly been proven useful in the treatment of various types of pathologies, defining thermalism as a natural treatment method synonymous with well-being [4]. The effectiveness of the thermal medicine has been highlighted, above all, in the management of pathologies affecting the musculoskeletal system [5,6] and the respiratory tract [7,8], thus proving to be a valid support approach for patients affected by these conditions. Also, in some of our previous research experiences, thermal medicine, in the form of Integrated Thermal Care (ITC) approach, has proven useful in multiple complex pathological contexts, showing benefits for neurological and musculoskeletal disabilities, up to the treatment of Long COVID-19 Syndrome [9,10], in which symptoms such as fatigue, pain and musculoskeletal functional limitations dominate the clinical picture of patients. The mechanisms through which balneotherapy could induce positive effects in terms of cardio-respiratory efficiency and tolerance to effort are multiple: it is believed that due to the high temperatures and mineral composition of spa waters, thermal medicine treatments can induce an improvement in blood pressure [11], an increase in oxygen transport to the tissues [12] and an efficiency of the respiratory organs due to mucolytic, pro-expectorant, anti-inflammatory and antioxidant activity (which is more intense for thermal waters containing sulfide and hydrosulfide ions) [13]. It is no coincidence that balneotherapy seems to be a promising approach to manage musculoskeletal and cardio-respiratory fatigue even in complex pathologies strongly characterized by reduced resistance to effort such as fibromyalgia [14], ankylosing spondylitis [15] and chronic stress syndromes [16].

Other situations in which fatigue, pain and reduced functional performance occur are, for example, the oncological and surgical contexts, especially when they overlap in the case of patients who have undergone surgery to remove tumors and consequently endure invasive pharmacological and surgical procedures, as often happens in women affected by breast cancer [17,18,19].

Furthermore, it must be considered that the symptoms mentioned are often aggravated by anxiety and depression that afflict mastectomized/quadrantectomized women [20]. In fact, a mastectomy/quadrantectomy can be disabling in terms of self-perception, further reducing the adherence of patients to rehabilitation protocols and consequently preventing a full recovery from physical symptoms [21,22].

The onset of pain, cardio-respiratory fatigue and functional limitation in cancer patients (especially in the operated shoulder–arm area) is frequently associated with the onset of an inflammatory state [23]. This inflammation could benefit from the direct anti-inflammatory and antioxidant effects associated with crenotherapy, which is intended as the therapeutic ingestion and breathing of thermal mineral waters: these effects have in fact been observed in multiple clinical contexts [24,25,26,27,28]. Furthermore, these symptoms would seem to benefit from treatments dedicated to the musculoskeletal system, such as the deactivation of the Myofascial Trigger Points (MTrPs) [29,30] that are established in the affected areas or exercise performed in water [31,32].

Although these treatment methods have been proven to be potentially effective in many pathological contexts, including oncology, to date, the literature is unable to provide a definitive answer on the eventual advantages offered by balneotherapy in the therapeutic field due to the heterogeneity of the treatments applied and studied [33,34,35].

Therefore, given the reduced invasiveness and high tolerability of treatments falling in the field of thermal medicine, paired with their potential effectiveness in reducing pain, physical limitations and cardiopulmonary deficits in a multitude of patients, we decided to conduct an observational study to verify if mastectomy/quadrantectomy patients who underwent a therapeutic course of thermal medicine according to the ITC approach, which combines therapeutic approaches such as crenotherapy, manual MTrPs stimulation and water exercise, tend to eventually obtain tangible benefits from the treatment in terms of a reduction in the pathological symptoms manifested.

## 2. Materials and Methods

### 2.1. Study Design and Ethics

This research is a pilot retrospective analytical observational study carried out from September to December 2023 at the “Castelnuovo della Daunia Thermal Medicine Center” (Castelnuovo della Daunia, Italy), affiliated with the national health system and the rehabilitation department of the Local Health Authority of Foggia (Italy), in cooperation with the staff of the “Center for Physiotherapy, Rehabilitation and Re-Education—Ce.Fi.R.R.” (Chieti, Italy).

The treatment protocol to which the patients were subjected is generally safe and not invasive with all the therapeutic procedures carried out complying with the safety regulations of the study country; the protocol is accessible to all patients who do not highlight specific contraindications to the initial clinical evaluation that is mandatory for all patients who access the study facility. The study has been performed in accordance with the Helsinki Declaration and Good Clinical Practice standards [36]; written informed consent was obtained at the initial clinical evaluation from all patients. Specifically, in accordance with the evaluation and operational routine applied at the study site, data considered for this study are those registered in the medical chart compiled by doctors in charge of the initial and follow-up evaluations of patients accessing the study facility [37]. Moreover, the research follows the general consensus according to which standard ethics committee approval is not required for non-pharmacological retrospective observational studies in the country where it was conducted [38]. Howsoever, the research project falls within the scope of the certification of technical and ethical suitability for the implementation of observational studies (certificate no. IT15/0304) issued to the “Ce.Fi.R.R.” institution, in charge of carrying out the study, by the Registered National Accreditation body ACCREDIA in accordance with the UNI EN ISO 9001:2015 regulation.

### 2.2. Sample

We considered data from 11 women (mean age 54 ± 6 years) recovering from mastectomy or quadrantectomy surgery for breast cancer to which they were subjected no less than 3 months before the start of the therapeutic protocol. The patients involved were all patients originating from the geographical area of reference of the study who after having undergone surgery for breast cancer spontaneously turned to the study site to undertake a non-invasive complementary treatment path. At initial clinical evaluation, carried out by specialist doctors in physical and rehabilitation medicine, rheumatology and pneumology, all patients presented symptoms of musculoskeletal pain, cardio-respiratory fatigue and functional limitation. Symptoms were verified by specialist doctors through an objective examination consisting of an interview with the patient, for the collection of subjective symptoms, coupled with an objective examination consisting of mobilization and palpation of the pathological areas (typically located in the upper quadrant of the body). The visit was then completed by a collection of quantitative data, consisting of the administration to the patient of tests and evaluation scales that make up the evaluation methods described below. Patients with severe cardiovascular diseases, neurological diseases and active infections were excluded from the study, since they are usually not considered suitable for undertaking a spa treatment. Any pharmacological support therapy followed by the patients during the observation period was agreed with the evaluating specialist doctors and kept stable throughout the observation period, thus trying to limit the influence of any variability in pharmacological therapy of each single patient on the effectiveness and tolerability of the proposed ITC treatments.

### 2.3. Assessment Methods

Patients were assessed, before the beginning (T0) and after the end (T1) of the pre-scribed ITC treatment cycle, using the following assessment tools:

- Piper Fatigue Scale (PIPER): The revised version of the PFS consists of 22 items and four subscales: behavioral/severity (6 items), affective meaning (5 items), sensory (5 items) and cognitive/mood (6 items). These items are rated using a 0–10 numeric scale. The 22 items are used to calculate the fatigue score. Five additional items are not used to calculate subscale or total fatigue scores but are recommended to be kept on the scale as these items furnish rich, qualitative data [39] (Figures S2–S4). This scale is also useful for assessing fatigue in oncology [40].
- 6-Minute Walking Test (6MWT): This tool consists of measuring the distance (expressed in meters) traveled in a time of 6 min by patients [41], allowing to evaluate the functional and cardiorespiratory fatigability of patients, also in the breast cancer field [41].
- Five Times Sit-to-Stand (5STS): This test consists of measuring the time (expressed in seconds) taken by the patient to sit down and stand up 5 consecutive times from a chair or raised surface; the test allows to evaluate lower extremity strength, dynamic balance, fall risks, gait speed and eventually fatigability [42].
- Numeric Pain Rating Scale (NPRS): It is one of the most widespread tools in clinical practice for assessing the pain subjectively perceived by patients. It is a derivate of the Visual Analogue Scale (VAS) divided into ten levels, usually distributed equidistant on a 10 cm long strip, which correspond to the level of pain perceived by the patient at the time of the evaluation. A value of 0 represents the total absence of pain, a value between 1 and 3 corresponds to mild pain, a value between 4 and 6 corresponds to moderate pain and a value between 7 and 10 corresponds to severe pain, where a score of 10 represents the maximum level of pain imaginable and/or ever experienced by the patient [43] (Figure S1).
- Disability of the Arm–Shoulder–Hand Scale (DASH): This is a self-administered questionnaire of over 30 questions designed to measure function and symptoms in patients with any musculoskeletal disorder specific to the upper limb. Questions address the ability to perform certain actions in the past week and symptoms that have occurred while performing these actions [44] (Figures S5 and S6). It is also highly reliable in assessing upper limb function in patients with mastectomy/quadrantectomy and breast cancer [44].
- Range of Arm Motion (ROM): The goniometric measurement (expressed in degrees) of the joint range of motion in the various planes of motion. In this case, the shoulder joint subjected to surgery was taken as a reference, considering flexion, extension, adduction, abduction, internal rotation and external rotation [45].

### 2.4. Treatment Methods

The ITC protocol administered to the study patients, performed for 1 h a day, 5 days a week for 4 weeks, consisted of a combination of the following therapeutic methods:

- Hydroponic Treatments: These correspond to the crenotherapy treatment. Initially, the bicarbonate–sulfate–alkaline–earthy mineral water mineral water from the “Castelnuovo della Daunia Thermal Medicine Center” was given to patients in the study facility, at the beginning of each treatment session, by drinking a total of 500 cc as desired on an empty stomach for at least 6 h before each treatment session. After receiving the water to drink, patients underwent steam inhalations treatment with direct warm humid jets placed in front of their face. The bicarbonate–sulfate–alkaline–earthy mineral water jets come out from a nozzle in the form of a homogeneous mist (pressure of 1.5 atmospheres—temperature of 37–38 °C), lasting about 20 min.
- Somatic stimulation: At the same time as the inhalations treatment, a contemporary manual somatic stimulation was performed by a physiotherapist on C1–C5 afferent areas of the neck of the patient for the whole 20 min of inhalation. This method consists of the digital pressure stimulation of the key MTrPs identified in the neck area of the patient, according to the Bio-Physico-Metric approach of hierarchical deactivation of the key and satellite MTrPs [46]. This method is based on palpation of the affected area in search of nodules in the muscle tissue, which are typically the site of MTrPs that can cause pain, functional limitation and possible irradiation symptoms that can also influence areas distant from the site of the MTrPs. Once the MTrPs of the anatomical area investigated have been identified, they are mechanically stimulated through pressure and massage techniques aimed at their resolution with cascading effects that have repercussions not only locally but also in the distant anatomical areas which are functionally influenced by the MTrPs defined as “key” [46]. In this way, it is possible to work on the tensions, pains and limitations of the cervical and shoulder areas as well as indirectly on further functions such as respiratory mechanics.
- Hydrokinesitherapy: Treatment in water environment, according to the Safe Bearing Back [47], was conducted with 40 min treatment sessions carried out in the bicarbonate–sulfate–alkaline–earthy mineral water pools of the study facility. For the first 20 min of the session, patients were treated by the physiotherapist through mobilization maneuvers and the deactivation of KMTrPs in the shoulder and trunk area while floating on the water surface supported by the same physiotherapist. The remaining 20 min of the treatment was dedicated to the execution of guided active exercises in water, in particular for the spine and upper limbs, including the use of ankle weights and hand-held plates useful for exploiting the hydraulic resistance offered by the water. The exercises were personalized and adapted according to the necessities and abilities of each patients, mainly using exercises of flexion, abduction, extension and internal and external rotations of the arms with the patient immersed in water up to the level of the neck as well as flexion and extension exercises of the lower limbs on the pelvis with the torso immersed in water.

### 2.5. Statistical Analysis

At the end of the study, statistical analysis was carried out on collected data using the Wilcoxon Signed Rank test for dependent samples, which was performed through the Statistics Kingdom online calculator (https://www.statskingdom.com/175wilcoxon_signed_ranks.html accessed on 3 September 2024, Melbourne, Australia).

## 3. Results

The following table shows the detailed demographic distribution of the sample considered for this study (Table 1).

**Figure 1:**
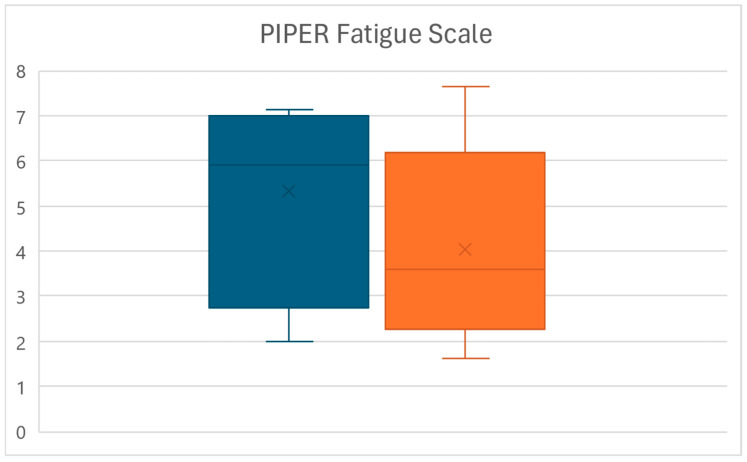
Box plots of the PIPER score variations between times T0 (left) and T1 (right).

At the end of the treatment protocol (T1), a significant reduction in the PIPER score value was observed (*p* < 0.01), with patients’ perception of pain decreasing from an average of 5.3 ± 1.9 to a mean of 4 ± 2.1, for an overall reduction of 24.3% (Figure 1).

**Figure 2:**
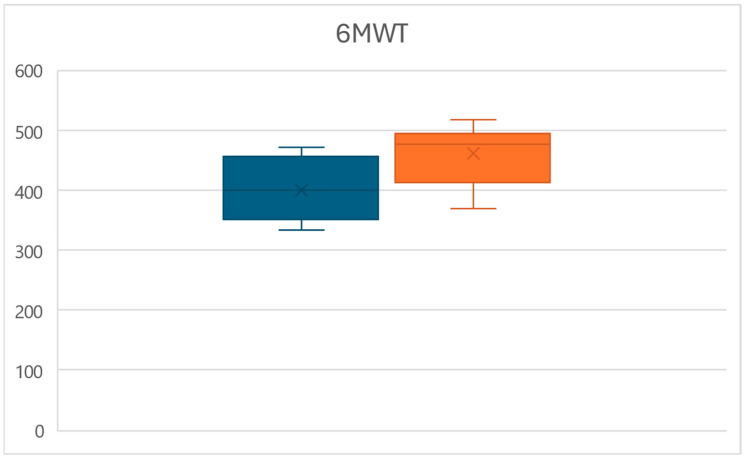
Box plots of the 6MWT distance variations between times T0 (left) and T1 (right).

Similarly, at time T1, a significant increase in the distance walked at the 6MWT was observed (*p* < 0.01), with values increasing from an average of 398.7 ± 49.6 m to a mean of 460.9 ± 48 m, for an overall increase of 15.6% (Figure 2).

**Figure 3:**
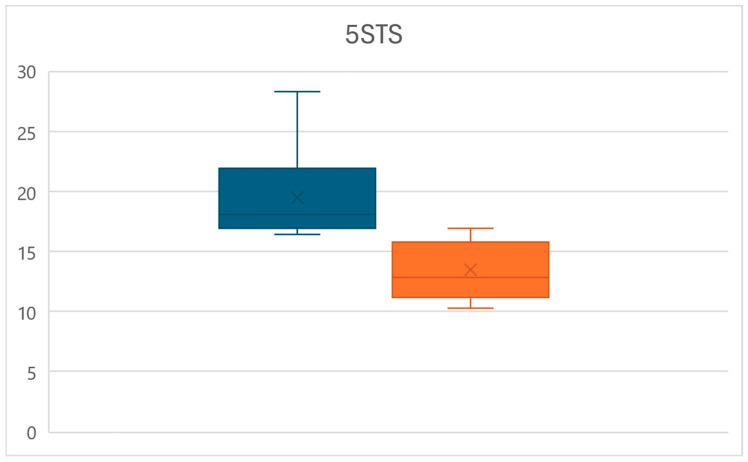
Box plots of the 5STS time variations between times T0 (left) and T1 (right).

At the same time, at time T1, a significant decrease in the time require to complete the 5STS test was observed (*p* < 0.01), with times decreasing from an average of 19.4 ± 3.5 s to a mean of 13.5 ± 2.3 s, for an overall reduction of 30.5% (Figure 3).

**Figure 4:**
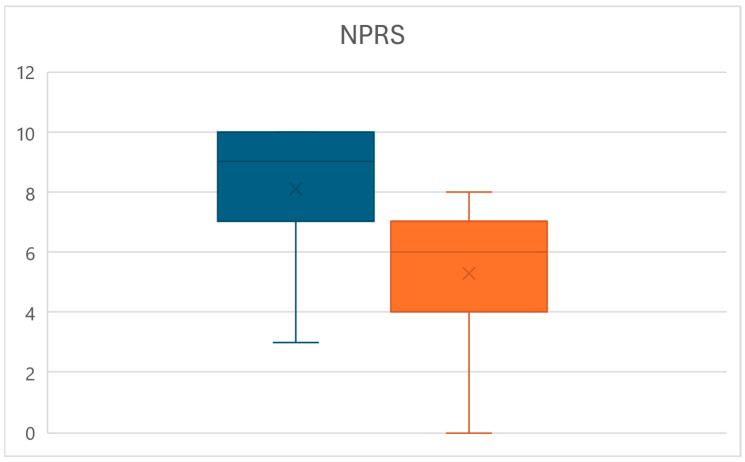
Box plots of the NPRS value variations between times T0 (left) and T1 (right).

Equally, at time T1, a significant reduction in the pain perceived by patients measured through the NPRS was observed (*p* < 0.01), with values decreasing from an average of 8.1 ± 2.2 to a mean of 5.3 ± 2.4, for an overall reduction of 34.8% (Figure 4).

**Figure 5:**
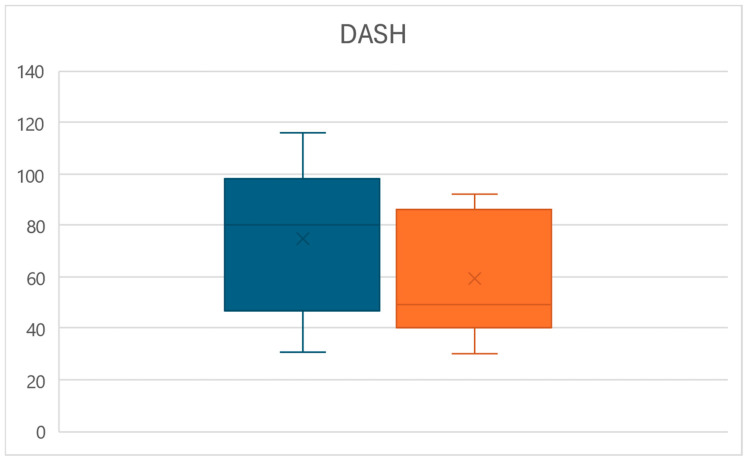
Box plots of the DASH score variations between times T0 (left) and T1 (right).

Likewise, at time T1, a significant decrease in the disability of the upper limb measured through the DASH score was observed (*p* < 0.01), with values reducing from an average of 74.6 ± 28.3 to a mean of 59.1 ± 23.8, for an overall reduction of 20.8% (Figure 5).

Lastly, slight increases in the joint ROM of the operated shoulder were observed at time T1 (Table 2), although these changes were significant only for external rotation (*p* < 0.02), with values reducing from an average of 69.1 ± 8.3 degrees to a mean of 73.6 ± 6.7, for an overall increase of 6.6% (Table 2).

## 4. Discussion

By analyzing the data collected at the end of the reference period considered, it was possible to observe how mastectomy/quadrantectomy patients who undergo a ITC-type balneotherapy protocol tend to show a significant improvement in all the parameters of resistance to fatigue, pain and functionality considered with the exception of the ROM of the shoulder on the side subjected to surgery.

Breast cancer and its clinical and surgical consequences represent a very important challenge today in the field of health promotion particularly in reference to the female population [48,49]. It is no coincidence that women are a population statistically more prone to the use of complementary and alternative medicine techniques to treat problems such as cancer, which can benefit from these treatment methods, as long as they do not completely replace ordinary medical treatments such as pharmacological, radiotherapy and surgery [50]. Among the most interesting and useful complementary and alternative medicine methods in many pathological fields, there is undoubtedly thermal medicine, which could have an important impact on the prevention and long-term treatment of pathologies that involve rather large sections of the population, such as oncological diseases [51]. Obviously, to better define the therapeutic potential of balneotherapy, it is necessary to investigate it from a scientific point of view in order to identify the most effective treatment protocols and healing patterns for the various categories of patients [52].

Thermal medicine is certainly an extremely complex field to investigate in the general oncology and breast cancer fields, although it would seem to have positive effects on factors such as pain, feelings of malaise and the general quality of life of patients, provided that it is applied with the necessary precautions relating to the specific pathological stage of the subjects treated [53].

A first aspect to analyze with respect to the therapeutic potential of thermal medicine is certainly that of the type of thermal water used for the treatments, where type means the specific mineral composition of thermal waters. Several studies have highlighted how the high mineralization of thermal waters contributes largely to their therapeutic effects in comparison to common oligomineral or non-mineralized waters [53,54]. It is in fact hypothesized that the chemical composition of thermal waters could have direct effects on the inflammatory and oxidative processes that are established in the presence of a chronic pathology [53,54] as it happens in the oncological field. In particular, bicarbonate–sulfate–alkaline–earthy mineral waters would seem to possess strong anti-inflammatory properties [55,56]. Since the water that flows at the thermal center where our study was carried out is precisely of the bicarbonate–sulfate–alkaline–earthy type, it is possible that the composition of the water has strongly contributed to the reduction in pain, fatigue and functional limitations observed in patients given its triple application by ingestion, inhalation and skin contact in our ITC protocol during the hydroponic treatments and hydrokinesitherapy phases.

Indeed, many studies suggest that inflammatory mechanisms play an important role in the genesis and perpetuation of pain and fatigue in cancer patients [57,58]. Moreover, the reduced level of physical activity induced by cancer treatments may contribute to further reduce cardiorespiratory compliance and physical efficiency in breast cancer patients [59]. Therefore, precisely by virtue of the coexistence of mechanisms linked to inflammation and reduced musculoskeletal efficiency in the genesis of the symptoms observed in patients, the ITC protocol combines the supplementation therapy with thermal waters with a direct somatic stimulation of a manual and motor type.

The somatic stimulations applied to the observed patients are part of a Bio-Physico-Metric approach aimed at identifying and dissolving key MTrPs present in the body of the patient [46]. In fact, key MTrPs can have an influence both locally and remotely through the genesis of a hierarchical and linked system of TrPs able to alter the musculoskeletal functionality of the patient [46]. It has been seen how MTrPs can be sites of accumulation of aberrant stimuli of a nociceptive and painful nature that can strongly influence the pain perception and physical functionality of the patient [60]. The formation of these MTrPs in breast cancer patients is exacerbated after mastectomy/quadrantectomy (particularly in the neck and shoulders) [24] and constitutes an important factor in peripheral and central sensitization to pain [61]. Furthermore, myofascial release applied to the neck, chest and diaphragm muscles, such as that implemented in the ITC protocol during MTrPs stimulations, would seem to be associated with a direct effect of improvement of cardio-respiratory parameters in pathological subjects [62], suggesting that the somatic stimulation of these areas could improve not only pain but also fatigue and musculoskeletal hypofunction. All this would seem to be confirmed by observations according to which the presence of active and latent MTrPs can determine the greater fatigability, pain and inefficiency of the muscles directly and indirectly affected by their aberrant activity [63]. Moreover, when muscle function is compromised due to MTrPs present at the temporomandibular, cervical and thoraco-dorsal levels, a chronic alteration of respiratory patterns can occur [64,65], with consequent impairment of the respiratory efficiency of patients, which can result in reduced fatigue tolerance and ability to cope with efforts [64]. Not surprisingly, some authors correlate the presence of alterations in respiratory patterns to the onset of chronic fatigue syndrome [66]. Furthermore, MTrPs can determine the onset of autonomic phenomena that induce a reduction in respiratory efficiency through the manifestation of symptoms such as cough or alteration of respiratory rhythm [67]. These assumptions are confirmed by the experiences of some authors who suggest that the stimulation of soft tissues, in the form of myofascial release, produces an increase in both thoracic mobility and spirometric respiratory parameters in breast cancer patients [68], which could contribute to the improvement of physical performance of patients subjected to this type of treatment. This concept of interrelationship between respiratory function and musculoskeletal alteration, particularly of the upper quadrant of the body, would seem to be reinforced by the correlation highlighted in other clinical contexts of reduced respiratory efficiency, such as asthma [69] or COPD [70].

Therefore, in light of all these considerations, we believe it is plausible that the combination of biochemical and somatic stimulations typical of the ITC approach may have largely contributed to the overall significant improvement in the monitored symptoms in the mastectomy/quadrantectomy patients who underwent the treatment protocol.

It must then be considered that the only parameters that in general, although improving, did not show significant variations regarding the ROM values of the dysfunctional shoulder. First of all, the reduced improvement should be contextualized to the fact that the ROM detected at T0 for the movements investigated was already within a physiological range or at least functional for the execution of a multitude of daily life tasks according to what has been identified in the literature [71]. This highlights a discrepancy between the disability subjectively perceived by patients through the DASH scale and the non-task-oriented mobility of the limb and reduces the possibility of highlighting large variations in the range of motion of the pathological limb. Furthermore, post-surgical scarring in breast cancer is a known factor in limiting the range of motion of the surgically incised areas, which in our case may have contributed to a relatively modest improvement in ROM [72]. In any case, it is important to note that the only significant improvement in ROM of the dysfunctional shoulder was observed for the external rotation movement, which is very important, considering that external rotation is one of the most limited movements following breast cancer surgery [73].

Obviously, it is appropriate to highlight some weaknesses of this study. First of all, the observational nature of the study has placed limits both in the selection of the sample and in the methods applied. The number of patients observed is in fact relatively small, as it refers only to women who voluntarily turned to the study site to undertake a course of complementary treatments such as the ITC protocol. This difficulty in recruitment is in line with what has been found in the literature as, to date, complementary and additional treatments in the oncological field are still rather undervalued, and it is not easy to convince patients or their oncologists to undertake a path such as, for example, the ITC proposed by us following problems related to oncological surgery or treatment [74]. This phenomenon is amplified by a general lack of knowledge among patients and referring clinicians of the usefulness of rehabilitative treatments in breast cancer survival [75]. It should also be noted that the study venue is located in a rather large and dispersed territory characterized by a lack of infrastructure typical of southern Italy [76], which may result in a further limitation in the accessibility to treatments for some patients in the area. Consequently, the aforementioned difficulties in collecting an adequate sample has also placed limits in terms of the homogeneity of the sample, which is widely variable in terms of age, type of surgical intervention undergone, type of supportive therapy performed, type of breast cancer and eventual comorbidities. These difficulties in sampling large and homogeneous groups of patients clearly also limited the possibility of making a comparison with a control group, also considering that at the study center, there were no data available for post-breast surgery oncology patients who had not undergone ITC treatment; in fact, the institutions involved in the study collect data useful for observations in the medical records of only those patients who voluntarily turn to the thermal medicine center to undertake a complementary and alternative therapeutic path. Furthermore, considering that the study location is a medical thermal center purely dedicated to clinical evaluations and rehabilitation treatments without sectors and instruments dedicated to the analysis of inflammatory or tumor markers or musculoskeletal and organ imaging, having to refer only to non-invasive and relatively quickly compiled tests, it was not possible to integrate more in-depth evaluation methods, such as blood tests, to have a clearer picture of the physiological effects of the treatments. On top of that, the observation period was relatively short since, considering that the study received no external funding, it was possible to observe patients only while undergoing treatments for the maximum number of sessions normally reimbursed to them by the national health system, according to the public nature of the healthcare system in force in the country where the study was conducted. However, most studies on balneotherapy tend to highlight an improvement in the symptoms taken into consideration in various clinical–pathological contexts with short-term protocols, which typically last 2–4 weeks, [16,77,78,79] consistently with the ITC protocol observed by us.

In any case, our study is a useful pilot to preliminarily investigate the efficacy of the observed treatment, as well as its safety, confirmed by the absence of drop-outs or adverse reactions in the sample considered. Any limitations may be taken as a warning and a starting point for improvement in the creation of new and more in-depth experimental studies on the topic, which should also include a possible control group and a long-term follow-up.

## 5. Conclusions

Based on our observations, the ITC thermal medicine protocol could determine multiple benefits in terms of pain, musculoskeletal and cardiorespiratory fatigue and functional limitation in patients undergoing surgery for the removal of breast cancer.

Since breast cancer poses great challenges for the future of medical and health sciences, the identification of effective, non-invasive therapeutic patterns that can accommodate a wide user base is of fundamental importance in the field of preventive medicine and long-term care.

Furthermore, the promotion of development and research activities in interesting fields such as thermal medicine can largely contribute to the growth of the level of interdisciplinary collaboration between oncologists, physiatrists, pulmonologists, rehabilitators and in general all medical and health professionals responsible for the health and well-being of patients.

Finally, it would be important to increase the level of knowledge and evidence regarding balneological treatments, as this would allow for better interfacing with local institutions to promote the large-scale integration and infrastructural improvement of treatment pathways dedicated to complex patients such as women undergoing breast removal surgery as in the case of the ITC treatment being studied.

## Figures and Tables

**Table 1:** Demographic distribution of the sample.

Variable	Categories	Frequencies
Total Patients	Women	11
Age (years)	31–45	1
	46–60	8
	61+	2
Body Mass Index	Normal (≥18.5 and <25)	4
	Overweight (≥25 and <30)	5
	Obese: Class I (≥30 and < 35)	1
	Obese: Class II (≥35 and < 40)	0
	Obese: Class III (≥40)	1
Cancer Side	Right	5
	Left	6
Cancer Type	Ductal in Situ	3
	Ductal Infiltrative	8
Surgery Type	Mastectomy	4
	Quadrantectomy	7
Radiotherapy	Yes	8
	No	3
Chemotherapy	Yes	6
	No	5

**Table 2:** ROM variations between times T0 (left) and T1 (right).

Variable	T0	T1	*p*-Value	Δ%
Flexion	166.4 ± 25°	170.9 ± 21.2°	n.s.	+2.7%
Extension	38.2 ± 5.1°	39.1 ± 4.4°	n.s.	+2.4%
Adduction	34.5 ± 6.5°	36.4 ± 4.5°	n.s.	+5.3%
Abduction	165.5 ± 25°	170.9 ± 20.7°	n.s.	+3.3%
Internal Rotation	88.2 ± 4°	89.1 ± 3°	n.s.	+1%
External Rotation	69.1 ± 8.3°	73.6 ± 6.7°	<0.02	+6.6%

n.s.: not significant.

## Data Availability

The data presented in this study are available upon reasonable and motivated request to the corresponding author due to privacy reasons.

## Supplementary Materials

The following supporting information can be downloaded at https://www.mdpi.com/article/10.3390/life15030374/s1, Figure S1: Italian version of the NPRS used during the study; Figures S2–S4: Italian version of the PIPER Scale used during the study; Figures S5 and S6: Italian version of DASH Scale used during the study.
