# Supplementary material for: Cardio-Respiratory, Functional and Antalgic Effects of the Integrated Thermal Care Protocol After Breast Cancer Surgery
Source: Life (Basel). 2025 Feb 27;15(3):374. doi: 10.3390/life15030374 (PMC11944069; doi:10.3390/life15030374)
Supplement: Supplementary file 1 [file life-15-00374-s001.zip › life-3470260-supplementary.pdf]

Figure S1: Italian version of the NPRS used during the study.

Terme di Castelnuovo della Daunia

Contrada Crocella, 71034, Castelnuovo della Daunia (FG)

Phone: 0881 559766 - Email: info@termedicastelnuovo.it

Scala di Valutazione del Dolore (Numeric Pain Rating Scale)

Paziente (Nome, Cognome e Data di Nascita)* ____

La linea orizzontale indicata di seguito presenta un intervallo che va da 0 a 10, corrispondenti rispettivamente a "nessun dolore" e "peggior dolore immaginabile". Il paziente è invitato ad indica l'intensità del proprio dolore, indicandola verbalmente all'esaminatore o facendo un segno sul numero che meglio lo descrive.

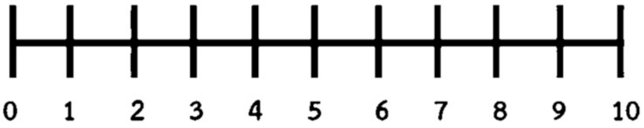

Data ______________________________

* La presente valutazione sarà custodita presso l'istituzione indicata in intestazione e trattata secondo le vigenti normative in materia di privacy e sicurezza dei dati esclusivamente per gli usi consentiti dal paziente.

Figure S2: Italian version of the PIPER Scale used during the study (page 1).

Terme di Castelnuovo della Daunia

Contrada Crocella, 71034, Castelnuovo della Daunia (FG)

Phone: 0881 559766 - Email: info@termedicastelnuovo.it

SCALA DI VALUTAZIONE DELLA FATICA (PIPER FATIGUE SCALE)

Paziente (Nome, Cognome e Data di Nascita)* ____

1) Da quanto tempo avverti questa sensazione di affaticamento? (Barrare una sola risposta).

1. non la sento
2. minuti
3. ore
4. giorni
5. settimane
6. mesi

7 Altro (specificare) ____

2) Fino a che punto avverti che è il senso di affaticamento che attualmente ti causa disagio?

Nessuno									Massimo
1	2	3	4	5	6	7	8	9	10

3) Fino a che punto avverti che è il senso di affaticamento che attualmente interferisce con la capacità di svolgere il tuo lavoro o le attività scolastiche?

Nessuno									Massimo
1	2	3	4	5	6	7	8	9	10

4) Fino a che punto avverti che è il senso di affaticamento che attualmente interferisce con la capacità di socializzare con i tuoi amici?

Nessuno									Massimo
1	2	3	4	5	6	7	8	9	10

5) Fino a che punto avverti che è il senso di affaticamento che attualmente interferisce con la capacità di avere una regolare attività sessuale?

Nessuno									Massimo
1	2	3	4	5	6	7	8	9	10

6) Nel complesso, fino a che punto avverti che è il senso di affaticamento che senti attualmente, che interferisce con la capacità di impegnarsi nel tipo di attività che più ti piace fare?

Nessuno									Massimo
1	2	3	4	5	6	7	8	9	10

7) Come definiresti l'intensità del senso di affaticamento che stai vivendo attualmente?

Lieve									Grave
1	2	3	4	5	6	7	8	9	10

8) Come definiresti l'intensità del senso di affaticamento che stai vivendo attualmente?

Piacevole									Spiacevole
1	2	3	4	5	6	7	8	9	10

Figure S3: Italian version of the PIPER Scale used during the study (page 2).

Terme di Castelnuovo della Daunia

Contrada Crocella, 71034, Castelnuovo della Daunia (FG)

Phone: 0881 559766 - Email: info@termedicastelnuovo.it

9) Come definiresti l'intensità del senso di affaticamento che stai vivendo attualmente?

Gradevole									Sgradevole
1	2	3	4	5	6	7	8	9	10

10) Come definiresti l'intensità del senso di affaticamento che stai vivendo attualmente??

Protettiva									Distruttiva
1	2	3	4	5	6	7	8	9	10

11) Come definiresti l'intensità del senso di affaticamento che stai vivendo attualmente??

Positiva									Negativa
1	2	3	4	5	6	7	8	9	10

12) Come definiresti l'intensità del senso di affaticamento che stai vivendo attualmente??

Normale									Anormale
1	2	3	4	5	6	7	8	9	10

13) Quanto ti senti ora:

Forte									Debole
1	2	3	4	5	6	7	8	9	10

14) Quanto ti senti ora:

Sveglio									Sonnolente
1	2	3	4	5	6	7	8	9	10

15) Quanto ti senti ora:

Vivace									Apatico
1	2	3	4	5	6	7	8	9	10

16) Quanto ti senti ora:

Vigoroso									Stanco
1	2	3	4	5	6	7	8	9	10

17) Quanto ti senti ora:

Energico									Fiacco
1	2	3	4	5	6	7	8	9	10

18) Quanto ti senti ora:

Paziente									Impaziente
1	2	3	4	5	6	7	8	9	10

20) Quanto ti senti ora:

Euforico									Depresso
1	2	3	4	5	6	7	8	9	10

21) Quanto ti senti ora:

Concentrato									Assente
1	2	3	4	5	6	7	8	9	10

Figure S4: Italian version of the PIPER Scale used during the study (page 3).

Terme di Castelnuovo della Daunia

Contrada Crocella, 71034, Castelnuovo della Daunia (FG)

Phone: 0881 559766 - Email: info@termedicastelnuovo.it

22) La tua memoria ora è:

Buona									Pessima
1	2	3	4	5	6	7	8	9	10

23) Reputi che i tuoi pensieri ora siano:

Chiari									Offuscati
1	2	3	4	5	6	7	8	9	10

24) In generale, cosa credi sia la causa o che possa contribuire direttamente alla tua sensazione di fatica?

____

____

25) In generale, la cosa migliore che hai trovato per alleviare la sensazione di fatica è:

____

____

26) C'è qualcos'altro che vorresti aggiungere per meglio descrivere la tua sensazione di fatica?

____

____

27) Stai vivendo altri sintomi in questo momento?

____

____

Interpretazione della PIPER FATIGUE SCALE

La Piper Fatigue Scale è composta da 22 item misurati da 0 a 10, che valutano quattro diverse dimensioni soggettive del senso di affaticamento: Gravità (6 item - da 2 a 7) ; significato (5 item: da 8 a 12); percezione (5 item: da 13 a 17); e umore (6 item: da 18 a 23). Questi 22 elementi vengono utilizzati per calcolare le quattro sottoscale e il punteggio complessivo del senso di affaticamento. Il questionario presenta cinque item aggiuntivi (1 e da 24 a 27) non utilizzati per calcolare i punteggi delle sottoscale o dell'affaticamento totale, ma utili per la raccolta di ulteriori informazioni. L'item 1, in particolare, fornisce un dato per valutare la durata del sintomo. Per la valutazione della Piper Fatigue Scale sommare i punteggi di tutti gli item di ogni specifica sottoscala e dividere per il numero degli item. Se avete dati mancanti ma l'intervistato ha risposto ad almeno il 75% -80% degli item, calcolate la media su di essi (es. 4 item completati: sommate i loro valori e dividete per 4) Per calcolare il punteggio totale della Piper Fatigue Scale: sommate i punteggi dei 22 item e dividete per 22. Interpretazione del senso di affaticamento: 0 Assente, 1-3 Lieve, 4-6 Moderato, 7-10 Grave.

Data ______________________________

* La presente valutazione sarà custodita presso l'istituzione indicata in intestazione e trattata secondo le vigenti normative in materia di privacy e sicurezza dei dati esclusivamente per gli usi consentiti dal paziente.

Figure S5: Italian version of DASH Scale used during the study (page 1).

Terme di Castelnuovo della Daunia

Contrada Crocella, 71034, Castelnuovo della Daunia (FG)

Phone: 0881 559766 - Email: info@termedicastelnuovo.it

SCALA DI VALUTAZIONE DELLA DISABILITA DI SPALLA, BRACCIO E MANO (DASH)

Paziente (Nome, Cognome e Data di Nascita)* ____

Istruzioni: Il presente questionario riguarda i Suoi sintomi e la Sua capacità di compiere alcune azioni. Risponda a ogni domanda facendo riferimento al Suo stato durante l'ultima settimana. Se non ha avuto l'opportunità di eseguire una delle azioni durante l'ultima settimana, risponda alla domanda provando a immaginare come avrebbe potuto eseguirla. Non importa con quale mano o braccio Lei esegue l'azione; risponda in base alla Sua capacità di compierla e senza tenere conto del modo in cui la compie.

Valuti la sua capacità di eseguire le seguenti azioni durante l'ultima settimana (indichi un numero).

	Nessuna difficoltà	Lieve difficoltà	Discreta difficoltà	Notevole difficoltà	Non ci sono riuscito
1. Svitare il coperchio di un barattolo ben chiuso o nuovo	1	2	3	4	5
2. Scrivere	1	2	3	4	5
3. Girare una chiave	1	2	3	4	5
4. Preparare un pasto	1	2	3	4	5
5. Aprire spingendo una porta pesante	1	2	3	4	5
6. Posare un oggetto su uno scaffale al di sopra della propria testa	1	2	3	4	5
7. Fare lavori domestici pesanti (es. lavare i pavimenti o i vetri)	1	2	3	4	5
8. Fare lavori di giardinaggio	1	2	3	4	5
9. Rifare il letto	1	2	3	4	5
10. Portare la borsa della spesa o una ventiquattrore	1	2	3	4	5
11. Portare un oggetto pesante (oltre 5 Kg)	1	2	3	4	5
12. Cambiare una lampadina posta al di sopra della propria testa	1	2	3	4	5
13. Lavarsi o asciugarsi i capelli	1	2	3	4	5
14. Lavarsi la schiena	1	2	3	4	5
15. Infilarsi un maglione	1	2	3	4	5
16. Usare un coltello per tagliare del cibo	1	2	3	4	5
17. Attività ricreative che richiedono poco sforzo (es. giocare a carte, lavorare a maglia)	1	2	3	4	5
18. Attività ricreative nelle quali si fa forza o si prendono colpi sul braccio, sulla spalla o sulla mano (es. usare il martello, giocare a tennis o a golf, ecc.)	1	2	3	4	5
19. Attività ricreative che richiedono un movimento libero del braccio (es. giocare a frisbee, a badminton, ecc.)	1	2	3	4	5
20. Far fronte alle necessità di spostamento (andare da un posto ad un altro)	1	2	3	4	5
21. Attività sessuale	1	2	3	4	5

Durante la settimana passata, in che misura il suo problema al braccio, alla spalla o alla mano ha interferito con le normali attività sociali con la famiglia, gli amici, i vicini di casa i gruppi di cui fa parte?

	Per nulla	Molto poco	Un po'	Molto	Moltissimo
22.	1	2	3	4	5

Durante la settimana passata è stato limitato nel suo lavoro o in altre attività quotidiane abituali a causa del suo problema al braccio, alla spalla o alla mano?

	Non mi ha limitato per nulla	Mi ha limitato leggermente	Mi ha limitato discretamente	Mi ha limitato molto	Non ci sono riuscito
23.	1	2	3	4	5

Figure S6: Italian version of DASH Scale used during the study (page 2).

Terme di Castelnuovo della Daunia

Contrada Crocella, 71034, Castelnuovo della Daunia (FG)

Phone: 0881 559766 - Email: info@termedicastelnuovo.it

Valuti l'intensità dei seguenti sintomi durante l'ultima settimana.

	Nessuno	Lieve	Discreto	Forte	Estremo
24. Dolore al braccio, alla spalla o alla mano	1	2	3	4	5
25. Dolore al braccio, alla spalla o alla mano nel compiere una qualsiasi attività specifica	1	2	3	4	5
26. Formicolio (sensazione di punture di spillo) al braccio, alla spalla o alla mano	1	2	3	4	5
27. Debolezza al braccio, alla spalla o alla mano	1	2	3	4	5
28. Rigidità del braccio, della spalla o della mano	1	2	3	4	5

Durante l'ultima settimana quanta difficoltà ha incontrato nel dormire a causa del dolore al braccio, alla spalla o alla mano?

	Nessuna difficoltà	Lieve difficoltà	Discreta difficoltà	Notevole difficoltà	Non ci sono riuscito
29.	1	2	3	4	5

Mi sento meno capace, meno fiducioso o meno utile a causa del mio problema al braccio, alla spalla o alla mano

	Non sono assolutamente d'accordo	Non sono d'accordo	Non saprei	Sono d'accordo	Sono assolutamente d'accordo
30.	1	2	3	4	5

MODULO LAVORATIVO (OPZIONALE)

Le seguenti domande si riferiscono all'impatto del suo problema al braccio, alla spalla o alla mano sul suo lavoro (compreso il lavoro in casa se questa è la sua attività principale). Indichi qual è il suo lavoro/attività: ________________________. Indichi su ogni riga il numero che meglio descrive la sua capacità fisica durante l'ultima settimana.

Ha avuto difficoltà:

	Nessuna difficoltà	Lieve difficoltà	Discreta difficoltà	Notevole difficoltà	Non ci sono riuscito
31. A utilizzare la Sua tecnica abituale per lavorare?	1	2	3	4	5
32. A svolgere il Suo lavoro abituale a causa del dolore al braccio, alla spalla o alla mano?	1	2	3	4	5
33. A fare il lavoro bene come vorrebbe?	1	2	3	4	5
34. A dedicare al Suo lavoro la consueta quantità di tempo?	1	2	3	4	5

MODULO ATTIVITÀ SPORTIVE/RICREATIVE (OPZIONALE)

Le seguenti domande si riferiscono all'impatto del Suo problema al braccio, alla spalla o alla mano sulla Sua capacità di suonare il Suo strumento musicale o praticare il Suo sport, o su entrambe le attività. Se pratica più di uno sport o suona più di uno strumento (o fa entrambe le cose) risponda facendo riferimento all'attività che è più importante per lei. Indichi quale sport o strumento è il più importante per lei: ________________________ . Indichi su ogni riga il numero che meglio descrive la sua capacità fisica durante l'ultima settimana.

Ha avuto difficoltà:

	Nessuna difficoltà	Lieve difficoltà	Discreta difficoltà	Notevole difficoltà	Non ci sono riuscito
35. A utilizzare la Sua tecnica abituale per suonare il suo strumento o praticare il suo sport?	1	2	3	4	5
36. A suonare il suo strumento o praticare il suo sport a causa del dolore al braccio, alla spalla o alla mano?	1	2	3	4	5
37. A suonare il suo strumento o praticare il suo sport bene come vorrebbe?	1	2	3	4	5
38. A dedicare al suo strumento o al suo sport la consueta quantità di tempo?	1	2	3	4	5

Data ________________________________

* La presente valutazione sarà custodita presso l'istituzione indicata in intestazione e trattata secondo le vigenti normative in materia di privacy e sicurezza dei dati esclusivamente per gli usi consentiti dal paziente.
